# Chaperonin containing t-complex polypeptide 1 subunit 6A correlates with lymph node metastasis, abnormal carcinoembryonic antigen and poor survival profiles in non-small cell lung carcinoma

**DOI:** 10.1186/s12957-020-01911-x

**Published:** 2020-07-06

**Authors:** Ting Zhang, Wang Shi, Ke Tian, Yushan Kong

**Affiliations:** Department of Pulmonary and Critical Care Medicine, Affiliated Nanhua Hospital, University of South China, 336 South of Dongfeng Road, Hengyang, 421000 China

**Keywords:** Chaperonin containing t-complex polypeptide 1 subunit 6A, Non-small cell lung carcinoma, Clinicopathological features, Disease-free survival, Overall survival

## Abstract

**Background:**

This study aimed to investigate the correlation of chaperonin containing t-complex polypeptide 1 subunit 6A (CCT6A) expression with clinicopathological features and survival profiles in non-small cell lung carcinoma (NSCLC) patients.

**Methods:**

A total of 381 NSCLC patients with Tumor, Node, Metastasis (TNM) stage I–IIIA who underwent tumor resection were retrospectively screened. Immunohistochemistry staining and semi-quantitative scoring were used to evaluate CCT6A expression in tumor and adjacent tissues. Clinicopathological features were retrieved. Disease-free survival (DFS) and overall survival (OS) were calculated.

**Results:**

CCT6A expression was elevated in tumor tissue (CCT6A high 47.5% vs. low 52.5%) compared with adjacent tissue (CCT6A high 30.4% vs. low 69.6%) (*P* < 0.001), and ROC curve displayed that CCT6A could distinguish tumor tissue from adjacent tissue. Moreover, tumor CCT6A high expression was associated with lymph node metastasis (*P* = 0.001), elevated TNM stage (*P* = 0.002), and abnormal carcinoembryonic antigen (*P* = 0.022). Kaplan–Meier curves displayed that tumor CCT6A high expression was negatively correlated with DFS and OS (all *P* < 0.001). Cox’s regression analysis disclosed that tumor CCT6A high expression independently predicted worse DFS (*P* < 0.001) (hazard ratio (HR) 1.659 (95% confidence interval (CI) 1.318–2.089)), and OS (*P* < 0.001) (HR 1.779 (95%CI 1.378–2.298)).

**Conclusions:**

CCT6A may present some clinical value in the management of NSCLC.

## Background

Non-small cell lung carcinoma (NSCLC) is the most common type of lung cancer (accounting for 80–85% of newly diagnosed lung cancers), whose incidence continues to rise during the last decades mainly due to the worsening environment and unfavorable lifestyle factors [1–4]. Regarding the cause of death in NSCLC patients, lung cancer is the predominant cause of death in the first 6 years after diagnosis, thereafter, lung cancer as the cause of death proportionally decreases with time since diagnosis (but remains over 30%), and cardiovascular diseases, as well as chronic obstructive pulmonary diseases (COPD), become the more important causes of death, especially for patients aged >60 years at diagnosis (up to 34% for cardiovascular diseases and up to 19% for COPD) [5]. Although the treatment for NSCLC has achieved great advances (including surgery, chemotherapy, targeted therapy, and immunotherapy), the majority of NSCLC patients still present tumor progression, metastasis as well as drug resistance, which lead to unsatisfactory prognosis (with a 5-year overall survival (OS) rate ranging from 15% to 25%) [6, 7]. Thus, seeking for biomarkers that could monitor NSCLC progression may help establishing individual treatment and further improve the prognosis of NSCLC patients.

The TCP1 ring complex (TRiC), also known as cytosolic chaperonin containing t-complex polypeptide 1 (CCT), is a kind of ATP-dependent molecular chaperonins that are responsible for the folding of nascent polypeptides [8]. TRiC consists of two back-to-back rings with eight paralogous subunits (α, β, γ, δ, ε, ζ-1 or ζ-2, η, and θ), which are accordingly encoded as TCP1, CCT2, CCT3, CCT4, CCT5, CCT6A or CCT6B, CCT7, and CCT8 respectively [9–11]. The eight subunits display high sequence homology, whereas the apical substrate-binding domains have quite divergent sequences between subunits [9]. All the subunits are necessary for the function of TRiC, meanwhile, each subunit shows a different contribution to cellular functions [12]. Noticeably, CCT6 contains two subunits: 6A and 6B [13]. CCT6B, as a molecular chaperone involved in protein folding mediated by cytoplasmic chaperonin containing TCP-1, is currently found to be highly expressed in the testis and mucosal wounds [14, 15]. As to CCT6A, a few recent data reveal that CCT6A promotes cell proliferation and metastasis in various cancers (such as NSCLC, colon carcinoma, and hepatocellular carcinoma (HCC)) [11, 16–20]. Besides, several clinical practices illuminate that CCT6A correlates with deteriorated tumor features and unsatisfactory prognosis in patients with several cancers (including breast cancer, HCC, and colorectal cancer). As for NSCLC patients, limited evidence is found, just two related existing studies: one literature shows that CCT6A is related to worse survival of NSCLC patients, but that previous finding is only supported by gene expression analysis in The Cancer Genome Atlas (TCGA) [19]; and another study suggests that CCT6A expression levels positively correlate with metastasis in patients with high TGF-β expression, while their findings are based on data collected from MSKCC NSCLC data sets [16].

Considering CCT6A was an important member of TRiC and had shown an oncogenic effect on cellular functions (including NSCLC), meanwhile, it was correlated with unfavorable progression and prognosis in cancer patients, we hypothesized that CCT6A might affect TRiC function and eventually correlated with disease progression in NSCLC patients, while no relative clinical research was done yet. Hence, our study enrolled 381 NSCLC patients to detect the expression of CCT6A in tumor and adjacent tissues and investigated the correlation of tumor CCT6A expression with clinicopathological features and survival profiles in these NSCLC patients.

## Methods

### Patients

This study retrospectively screened 381 NSCLC patients who were admitted to our hospital and received surgical resection from January 2012 to December 2014. The inclusion criteria were (1) diagnosed as primary NSCLC by clinical and histopathological examinations according to the New World Health Organization Classification of Lung Tumors [21]; (2) age over 18 years; (3) tumor and adjacent tissue derived from primary surgery were available for study use; (4) detailed clinical feature data and complete follow-up data. The exclusion criteria were (1) received neoadjuvant therapy before surgery; (2) relapsed NSCLC or advanced (Tumor, Node, Metastasis (TNM) stage IIIB/IV) NSCLC confirmed by pathological assessment and computed tomography (CT) examination; (3) had history of other tumors or malignant hematological diseases. The approval for this study was obtained from Institutional Review Board of our hospital, and the written informed consents or verbal informed consents with tape recording were collected from patients or their family members. The detailed information of study design and patient’s data was displayed in the Supplementary Figure 1.

### Data extraction

By reviewing the electronic medical records, patients’ basic clinical features were collected, including age, gender, history of smoke, history of drink, complications (hypertension, hyperlipidemia, diabetes), pathological differentiation confirmed by pathological examination, tumor size, lymph node (LYN) status, TNM stage according to the American Joint Committee on Cancer (AJCC) 7th Edition Cancer Staging Manual, as well as carcinoembryonic antigen (CEA) level. In addition, survival data were collected from follow-up records with the last follow-up date being 2019/12/31.

### Immunohistochemistry (IHC) staining

Formalin-fixed paraffin-embedded (FFPE) tumor and adjacent tissue specimens were collected from the Pathology Department of our hospital. IHC staining was performed to evaluate the expression of CCT6A on 4-μm thickness FFPE tissue sections. The rabbit Anti-CCT6A antibody (Abcam, USA) was used as the primary antibody at 1:100 dilution, and the Goat Anti-Rabbit IgG H&L (HRP) (Abcam, USA) was used as secondary antibody at 1:50000 dilution. In brief, the sections were deparaffinized and hydrated. The heat-induced antigen epitope retrieval was performed using the microwave method, and the sections were immersed in EDTA antigen retrieval solution for 20 min. Subsequently, 3% hydrogen peroxide was added to inhibit endogenous peroxidase activity. Then sections were incubated with primary antibody at 4 °C overnight. The next day, sections were incubated with a secondary antibody at 37 °C for 60 min. The diaminobenzidine (DAB) (Sigma-Aldrich, USA) and hematoxylin (Sigma-Aldrich, USA) were used for staining and counterstain. After sealed with neutral tree gum sequentially, sections were prepared for visualization under light microscope (Olympus Corp, Japan).

### CCT6A expression assessment

A semi-quantitative scoring method based on the average intensity and percentage of positively stained tumor cells was used to assess the CCT6A expression in the tumor and adjacent tissue sections. Details of IHC scoring procedures were described in a previous study [22], and the total IHC score ranged from 0 to 12. IHC score ≤ 3 was defined as CCT6A low expression, correspondingly, IHC score between 4 and 12 was defined as CCT6A high expression, correspondingly. For survival analysis, the CCT6A high expression was further classified as high+ (IHC score 4–6), high++ (IHC score 7–9), and high+++(IHC score 10–12).

### Adjuvant therapy and follow-up

After initial surgical resection, patients were given surveillance, secondary resection or adjuvant therapy, according to the surgical margin status. For patients with negative surgical margin, surveillance or chemotherapy with cisplatin-based regimens was administered for them. As for patients with positive surgical margin, secondary resection or chemoradiotherapy (50~74 Gy with cisplatin and paclitaxel) was conducted for them. In addition, all patients were followed up regularly. The medical history, physical examination, and chest-enhanced CT were performed every 4–6 months for 2 years, then a history of the disease, physical examination, and non-enhanced chest CT were performed annually. The median follow-up duration of all patients was 57.0 months ranging from 2.0 to 94.0 months.

### Statistical analysis

SPSS version 22.0 (IBM, USA) was used for statistical analyses, and figures were plotted using GraphPad Prism version 7.00 (GraphPad Software, USA). Data were expressed as mean±standard deviation (SD), median and interquartile range (IQR), and count (percentage). The difference in CCT6A expression between tumor and adjacent tissue was determined by McNemar’s test. The difference of CCT6A IHC score between tumor and adjacent tissue was determined by the paired *t* test. The comparison of clinical features between CCT6A high expression patients and CCT6A low expression patients was determined by the chi-square test or Wilcoxon rank-sum test. The ability of CCT6A expression in discriminating NSCLC tissue from adjacent tissue was assessed by the receiver-operating characteristic (ROC) curve with sensitivity and specificity at the best cut-off point. Disease-free survival (DFS) was calculated from the date of surgery to the date of disease relapse, progression, or death. Overall survival (OS) was calculated from the date of surgery to the date of death. Both DFS and OS were displayed using Kaplan–Meier curve, and the comparison of DFS and OS between/among groups was determined by the log-rank test. All clinical features (listed in Table 1) were included in the univariate Cox’s proportional hazard regression model analysis to screen the factors predicting DFS and OS, while only factors with *P* < 0.05 in the univariate Cox’s proportional hazard regression model analysis were further included in the forward stepwise multivariate Cox’s proportional hazard regression model analysis. *P* value <0.05 was considered significant.

**Table 1:** Clinical features of NSCLC patients

Items	NSCLC patients (*N* = 381)
Age (years), mean±SD	61.0 ± 10.3
Gender (male/female), No.	286/95
History of smoke, No. (%)	208 (54.6)
History of drink, No. (%)	150 (39.4)
Common complications, No. (%)	
Hypertension	146 (38.3)
Hyperlipidemia	120 (31.5)
Diabetes	62 (16.3)
Differentiation, No. (%)	
Well	56 (14.7)
Moderate	228 (59.8)
Poor	97 (25.5)
Tumor size (cm), mean±SD	5.2 ± 2.1
LYN metastasis, No. (%)	134 (35.2)
TNM stage, No. (%)	
I	131 (34.4)
II	107 (28.1)
III	143 (37.5)
CEA (ng/mL), median (IQR)	6.6 (3.2–27.1)

*NSCLC* non-small cell lung carcinoma; *SD* standard deviation; *LYN* lymph node; *CEA* carcinoembryonic antigen; *IQR* interquartile range

## Results

### Clinical features of NSCLC patients

A total of 381 NSCLC patients with a mean age of 61.0 ± 10.3 years were analyzed in this study, including 286 males and 95 females (Table 1). There were 208 (54.6%) patients and 150 (39.4%) patients with a history of smoke and history of drinking, respectively. Meanwhile, 146 (38.3%), 120 (31.5%), and 62 (16.3%) patients had hypertension, hyperlipidemia, and diabetes respectively. Regarding tumor features, 56 (14.7%), 228 (59.8%), and 97 (25.5%) patients presented with well differentiation, moderate differentiation, and poor differentiation, respectively; besides, the mean tumor size was 5.2 ± 2.1 cm; and there were 134 (35.2%) patients showed LYN metastasis; as for the TNM stage, 131 (34.4%), 107 (28.1%), and 143 (37.5%) patients were with TNM stage I, TNM stage II, and TNM stage III, respectively. Additionally, the median CEA was 6.6 (3.2–27.1) ng/mL.

### CCT6A expression in tumor tissue and adjacent tissue in NSCLC patients

**Fig. 1:**
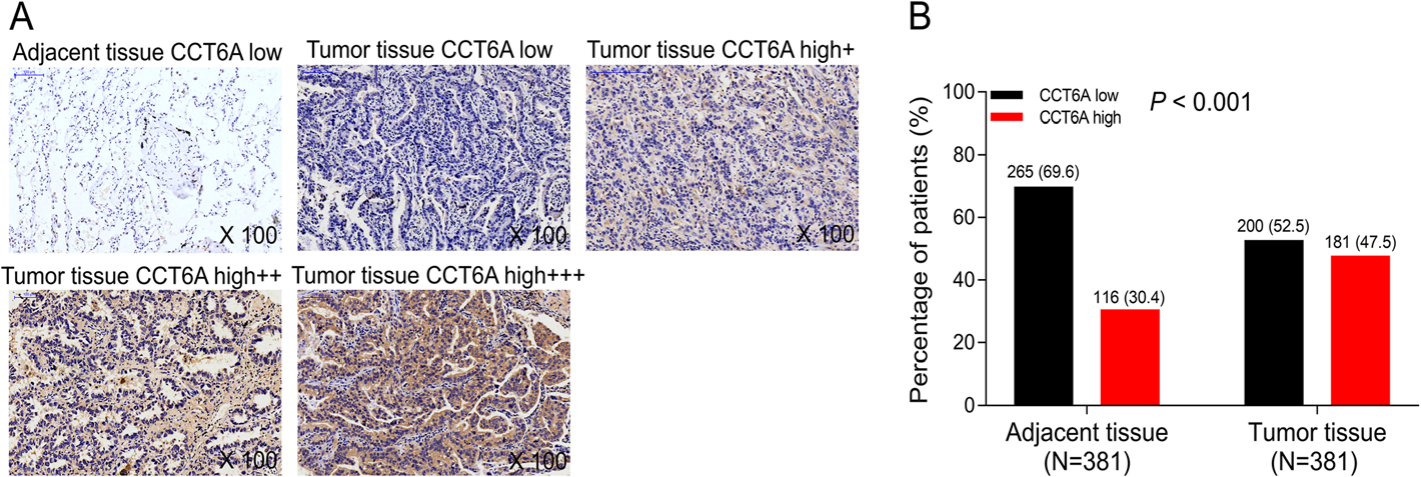
Expression of CCT6A in tumor tissue and adjacent tissue in NSCLC patients. Examples of adjacent tissue CCT6A low expression, tumor tissue CCT6A low expression, tumor tissue CCT6A high+ expression, tumor tissue CCT6A high++ expression, tumor tissue CCT6A high +++ expression (**a**). Comparison of CCT6A expression between tumor tissue and adjacent tissue (**b**). CCT6A, chaperonin containing t-complex polypeptide 1 subunit 6A; NSCLC, non-small cell lung carcinoma

CCT6A expression in tumor tissue and adjacent tissue was detected by IHC, and the examples of CCT6A expression in tumor and adjacent tissues were shown in Fig. 1a, which displayed that CCT6A mainly expressed in the cytoplasm of cells. Also, we observed that CCT6A expression was elevated in tumor tissue compared with adjacent tissue (*P* < 0.001) (Fig. 1b). Moreover, IHC score was used to semi-quantitatively determine the CCT6A expression, which showed that CCT6A IHC score was increased in tumor tissue than adjacent tissue (Supplementary Figure 2A), and ROC curves displayed that CCT6A could discriminate NSCLC tissue from adjacent tissue (area under the curve (AUC) 0.708, 95%CI 0.671–0.744) (Supplementary Figure 2B). Additionally, the best cut-off point was at IHC score of 1.5, with the sensitivity and specificity of 76.1% and 53.5% respectively; at the point that IHC score = 4, CCT6A showed the ability to distinguish NSCLC tissue from adjacent tissue with the sensitivity and specificity of 47.5% and 69.6% respectively (Supplementary Figure 2B).

### Association of tumor CCT6A expression with clinicopathological features in NSCLC patients

Tumor CCT6A high expression was associated with LYN metastasis (*P* = 0.001), elevated TNM stage (*P* = 0.002), and abnormal CEA (*P* = 0.022) (Table 2), while no correlation of tumor CCT6A expression with age (*P* = 0.239), gender (*P* = 0.458), history of smoke (*P* = 0.107), history of drink (*P* = 0.565), hypertension (*P* = 0.774), hyperlipidemia (*P* = 0.824), diabetes (*P* = 0.479), differentiation (*P* = 0.627), or tumor size (*P* = 0.106) was observed in NSCLC patients.

**Table 2:** Correlation of tumor CCT6A expression with clinical features

Items	Tumor CCT6A expression		*P* value
	Low (*n* = 200)	High (*n* = 181)	
Age, No. (%)			0.239
≤60.0 years	106 (53.0)	85 (47.0)	
>60.0 years	94 (47.0)	96 (53.0)	
Gender, No. (%)			0.458
Female	53 (26.5)	42 (23.2)	
Male	147 (73.5)	139 (76.8)	
History of smoke, No. (%)			0.107
No	83 (41.5)	90 (49.7)	
Yes	117 (58.5)	91 (50.3)	
History of drink, No. (%)			0.565
No	124 (62.0)	107 (59.1)	
Yes	76 (38.0)	74 (40.9)	
Hypertension, No. (%)			0.774
No	122 (61.0)	113 (62.4)	
Yes	78 (39.0)	68 (37.6)	
Hyperlipidemia, No. (%)			0.824
No	136 (68.0)	125 (69.1)	
Yes	64 (32.0)	56 (30.9)	
Diabetes, No. (%)			0.479
No	170 (85.0)	149 (82.3)	
Yes	30 (15.0)	32 (17.7)	
Differentiation, No. (%)			0.627
Well	35 (17.5)	21 (11.6)	
Moderate	112 (56.0)	116 (64.1)	
Poor	53 (26.5)	44 (24.3)	
Tumor size, No. (%)			0.106
≤5.0 cm	130 (65.0)	103 (56.9)	
>5.0 cm	70 (35.0)	78 (43.1)	
LYN metastasis, No. (%)			0.001
No	145 (72.5)	102 (56.4)	
Yes	55 (27.5)	79 (43.6)	
TNM stage, No. (%)			0.002
I	82 (41.0)	49 (27.1)	
II	55 (27.5)	52 (28.7)	
III	63 (31.5)	80 (44.2)	
CEA, No. (%)			0.022
Normal (≤5.0 ng/mL)	95 (47.5)	65 (35.9)	
Abnormal (>5.0 ng/mL)	105 (52.5)	116 (64.1)	

Comparison was determined by chi-square test or Wilcoxon rank-sum test. *CCT6A* chaperonin containing TCP1 subunit 6A; *LYN* lymph node; *CEA* carcinoembryonic antigen

### Association of tumor CCT6A expression with DFS in NSCLC patients

**Fig. 2:**
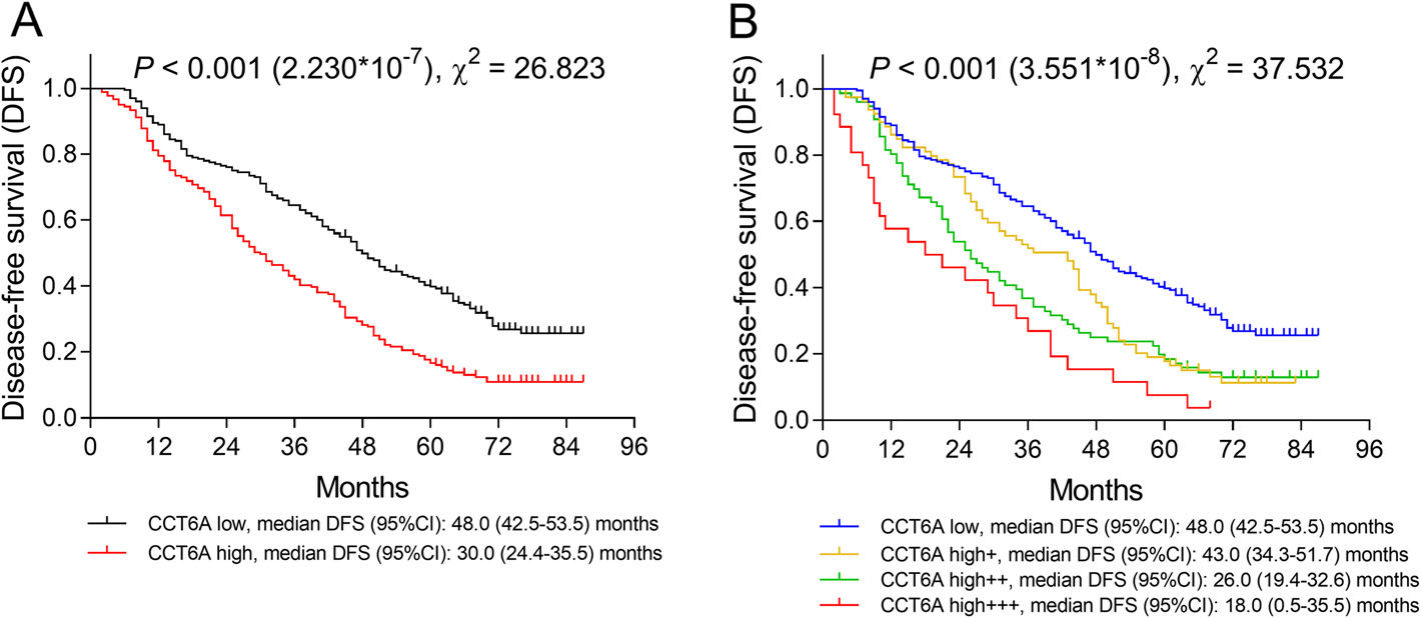
Negative correlation of DFS with CCT6A expression in NSCLC patients. Comparison of DFS between CCT6A high expression patients and CCT6A low expression patients (**a**). Comparison of DFS among CCT6A high +++ expression patients, CCT6A high++ expression patients, CCT6A high+ expression patients and CCT6A low expression patients (**b**). DFS, disease-free survival; CCT6A, chaperonin containing t-complex polypeptide 1 subunit 6A; NSCLC, non-small cell lung carcinoma

The median follow-up duration of all patients was 57.0 months ranging from 2.0 to 94.0 months. Kaplan–Meier (K-M) curves showed that tumor CCT6A high expression was associated with decreased DFS (*P* < 0.001, χ^2^ = 26.823): the median DFS was 30.0 (95%CI 24.4–35.5) months in CCT6A high expression patients and was 48.0 (95%CI 42.5–53.5) months in CCT6A low expression patients (Fig. 2a). In addition, patients with CCT6A high+++ expression showed the shortest DFS (median 18.0 (95%CI 0.5–35.5) months), followed by patients with CCT6A high++ expression (median 26.0 (95%CI 19.4–32.6) months), patients with CCT6A high+ expression (median 43.0 (95%CI 34.3–51.7) months), and patients with CCT6A low expression presented with the longest DFS (*P* < 0.001, χ^2^ = 37.532) (Fig. 2b). Besides, some other characteristics including poor differentiation, LYN metastasis, higher TNM stage, and abnormal CEA level were also correlated with shorter DFS (Supplementary Figure 3); regarding the statistical value (χ^2^) underlying K-M curve of these biomarkers, CCT6A showed a better predictive ability for worse DFS compared with poor differentiation (*P* = 0.001, χ^2^ = 13.395) (Supplementary Figure 3A) and abnormal CEA (*P* = 0.002, χ^2^ = 9.665) (Supplementary Figure 3D) and was inferior in predicting worse PFS compared with LYN metastasis (*P* < 0.001, χ^2^ = 56.599) (Supplementary Figure 3B) and higher TNM stage (*P* < 0.001, χ^2^ = 30.279) (Supplementary Figure 3C) in NSCLC patients.

### Association of tumor CCT6A expression with OS in NSCLC patients

**Fig. 3:**
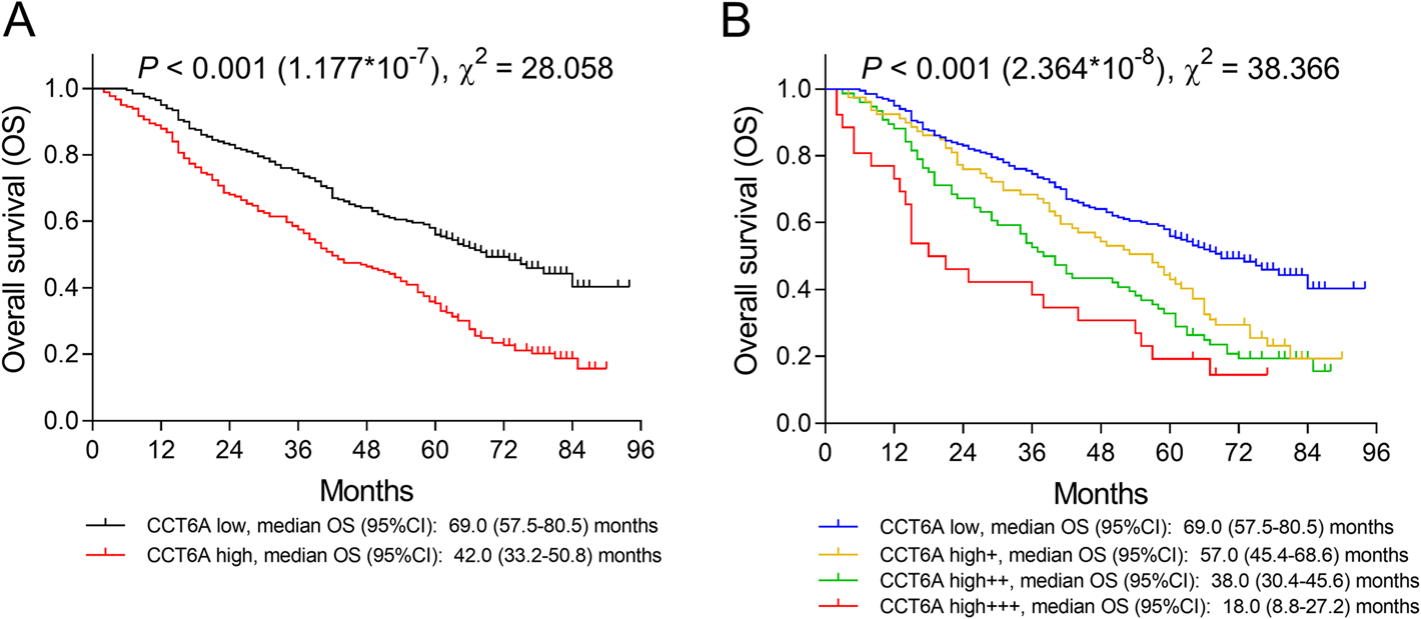
Negative correlation of OS with CCT6A expression NSCLC patients. Comparison of OS between CCT6A high expression patients and CCT6A low expression patients (**a**). Comparison of OS among CCT6A high +++ expression patients, CCT6A high++ expression patients, CCT6A high+ expression patients and CCT6A low expression patients. OS, overall survival; NSCLC, non-small cell lung carcinoma; CCT6A, chaperonin containing t-complex polypeptide 1 subunit 6A

K-M curves showed that tumor CCT6A high expression was associated with reduced OS (*P* < 0.001, χ^2^ = 28.058): the median OS was 42.0 (95%CI 33.2–50.8) months in CCT6A high expression patients and was 69.0 (95%CI 57.5–80.5) months in CCT6A low expression group (Fig. 3a). Besides, patients with CCT6A high+++ expression had the worst OS (median 18.0 (95%CI 8.8–27.2) months), followed by patients with CCT6A high++ expression (median 38.0 (95%CI 30.4–45.6) months), patients with CCT6A high+ expression (median 57.0 (95%CI 45.4–68.6) months), and patients with CCT6A low expression showed the best OS (*P* < 0.001, χ^2^ = 38.366) (Fig. 3b). Additionally, poor differentiation, LYN metastasis, higher TNM stage, and abnormal CEA level were also correlated with worse OS in NSCLC patients (Supplementary Figure 3); according to the statistical value (χ^2^) of K-M curve underlying these biomarkers, CCT6A had a better predictive ability for shorter OS compared with poor differentiation (*P* < 0.001, χ^2^ = 19.343) (Supplementary Figure 3E), higher TNM stage (*P* < 0.001, χ^2^ = 24.597) (Supplementary Figure 3G), and abnormal CEA level (*P* < 0.001, χ^2^ = 23.006) (Supplementary Figure 3H), but was relatively weaker in predicting shorter OS compared with LYN metastasis (*P* < 0.001, χ^2^ = 82.812) (Supplementary Figure 3F) in NSCLC patients.

### Analysis of factors affecting DFS in NSCLC patients

Univariate Cox’s regression showed that tumor high CCT6A was associated with decreased DFS (*P* < 0.001, HR 1.802 (95%CI 1.434–2.265)), meanwhile, age (>60.0 years) (*P* = 0.019, HR 1.314 (95%CI 1.047–1.649)), poor differentiation (*P* = 0.002, HR 1.306 (95%CI 1.102–1.548)), tumor size (>5.0 cm) (*P* = 0.008, HR 1.363 (95%CI 1.083–1.715)), LYN metastasis (*P* < 0.001, HR 2.383 (95%CI 1.883–3.016)), higher TNM stage (*P* < 0.001, HR 1.448 (95%CI 1.265–1.658)), and abnormal CEA (>5.0 ng/mL) (*P* = 0.002, HR 1.434 (95%CI 1.138–1.806)) were correlated with shorter DFS as well (Table 3). Moreover, multivariate Cox’s regression showed that tumor CCT6A high expression (*P* < 0.001, HR 1.659 (95%CI 1.318–2.089)) independently predicted poor DFS, meanwhile, poor differentiation (*P* = 0.006, HR 1.281 (95%CI 1.073–1.530)), LYN metastasis (*P* < 0.001, HR 2.227 (95%CI 1.757–2.824)) as well as abnormal CEA (>5.0 ng/mL) (*P* = 0.004, HR 1.405 (95%CI 1.114–1.772)) were independent predictive factors for worse DFS.

**Table 3:** Factors predicting DFS

Items	Cox’s proportional hazard regression model			
	*P* value	HR	95%CI	
			Lower	Higher
Univariate Cox’s regression				
CCT6A high	<0.001	1.802	1.434	2.265
Age (>60.0 years)	0.019	1.314	1.047	1.649
Male	0.064	1.297	0.985	1.708
History of smoke	0.326	0.892	0.711	1.120
History of drink	0.347	1.117	0.887	1.405
Hypertension	0.386	0.902	0.714	1.139
Hyperlipidemia	0.823	0.973	0.762	1.241
Diabetes	0.363	0.864	0.631	1.183
Poor differentiation	0.002	1.306	1.102	1.548
Tumor size (>5.0 cm)	0.008	1.363	1.083	1.715
LYN metastasis	<0.001	2.383	1.883	3.016
Higher TNM stage	<0.001	1.448	1.265	1.658
Abnormal CEA (>5.0 ng/mL)	0.002	1.434	1.138	1.806
Forward stepwise multivariate Cox’s regression				
CCT6A high	<0.001	1.659	1.318	2.089
Poor differentiation	0.006	1.281	1.073	1.530
LYN metastasis	<0.001	2.227	1.757	2.824
Abnormal CEA (>5.0 ng/mL)	0.004	1.405	1.114	1.772

Factors predicting DFS were analyzed by univariate Cox’s proportional hazard regression models. The factors with *P* < 0.05 in univariate Cox’s regression were further analyzed in forward stepwise multivariate Cox’s regression. *DFS* disease-free survival; *HR* hazard ratio; *CI* confidence interval; *CCT6A* chaperonin containing TCP1 subunit 6A; *LYN* lymph node; *CEA* carcinoembryonic antigen

### Analysis of factors affecting OS in NSCLC patients

Univariate Cox’s regression disclosed that tumor CCT6A high expression (*P* < 0.001, HR 1.952 (95%CI 1.515–2.516)) was correlated with unfavorable OS, and poor differentiation (*P* = 0.001, HR 1.368 ((95%CI 1.133–1.652)), tumor size (>5.0 cm) (*P* < 0.001, HR 1.605 (95%CI 1.248–2.064)), LYN metastasis (*P* < 0.001, HR 3.081 (95%CI 2.385–3.979)), higher TNM stage (*P* < 0.001, HR 1.446 (95%CI 1.244–1.682)) as well as abnormal CEA (>5.0 ng/mL) (*P* < 0.001, HR 1.880 (95%CI 1.444–2.447)) were also correlated with worse OS (Table 4). Furthermore, based on multivariate Cox’s regression analysis, we found that tumor CCT6A high expression (*P* < 0.001, HR 1.779 (95%CI 1.378–2.298)) was an independent predictive factor for predicting poor OS, and poor differentiation (*P* = 0.003, HR 1.360 (95%CI 1.113–1.663)), LYN metastasis (*P* < 0.001, HR 2.825 (95%CI 2.183–3.656)) as well as abnormal CEA (>5.0 ng/mL) (*P* < 0.001, HR 1.816 (95%CI 1.393–2.368)) independently predicted shorter OS in NSCLC patients as well.

**Table 4:** Factors predicting OS

Items	Cox’s proportional hazard regression model			
	*P* value	HR	95%CI	
			Lower	Higher
Univariate Cox’s regression				
CCT6A high	<0.001	1.952	1.515	2.516
Age (>60.0 years)	0.156	1.199	0.933	1.541
Male	0.657	1.069	0.797	1.432
History of smoke	0.149	0.832	0.648	1.068
History of drink	0.460	1.101	0.853	1.421
Hypertension	0.454	0.906	0.698	1.174
Hyperlipidemia	0.590	0.928	0.706	1.219
Diabetes	0.308	0.834	0.588	1.183
Poor differentiation	0.001	1.368	1.133	1.652
Tumor size (>5.0 cm)	<0.001	1.605	1.248	2.064
LYN metastasis	<0.001	3.081	2.385	3.979
Higher TNM stage	<0.001	1.446	1.244	1.682
Abnormal CEA (>5.0 ng/mL)	<0.001	1.880	1.444	2.447
Forward stepwise multivariate Cox’s regression				
CCT6A high	<0.001	1.779	1.378	2.298
Poor differentiation	0.003	1.360	1.113	1.663
LYN metastasis	<0.001	2.825	2.183	3.656
Abnormal CEA (>5.0 ng/mL)	<0.001	1.816	1.393	2.368

Factors predicting OS were analyzed by univariate Cox’s proportional hazard regression models. The factors with *P* < 0.05 in univariate Cox’s regression were further analyzed in forward stepwise multivariate Cox’s regression. *OS* overall survival; *HR* hazard ratio; *CI* confidence interval; *CCT6A* chaperonin containing TCP1 subunit 6A; *LYN* lymph node; *CEA* carcinoembryonic antigen

## Discussion

CCT is known about the complex interplay with actin and tubulin. For instance, the CCT oligomer folds newly synthesized tubulin and actin and also affects both actin transcription and assembled actin filaments; CCT behaves as microtubule-associated proteins and plays a role in dynein-mediated transport along microtubules [9]. Thus, CCT intrinsically connects to all cellular processes that rely on the microtubule and actin filament components of the cytoskeleton [9, 23]. Some recent studies uncover the role of CCT6A in tumor pathology [17, 18, 24, 25]. For instance, CCT6A is upregulated in drug-resistant variants of the human melanoma cell line compared with the parental human melanoma cell line [25]. Besides, CCT6A promotes cell proliferation through accelerating the G1-to-S transition in HCC [18]. Also, CCT6A knockdown dramatically decreases the proliferation of colon carcinoma cells [17]. As for lung cancer, one experiment displays that CCT6A enhances NSCLC cell metastasis [16]. These data reveal that CCT6A presents with oncogenic effect via promoting cell proliferation, enhancing G1-to-S transition, or facilitating drug resistance in specific cancers, including NSCLC.

In clinical practices, CCT6A has been shown to be dysregulated in cancer patients [18, 26]. For example, CCT6A is upregulated in breast cancer tissue, HCC tissue, and colorectal cancer tissue compared with the matched noncancerous tissue [17, 18, 26]. Furthermore, CCT6A high expression correlates with elevated clinical stage, larger tumor size, nodal statu,s and Scarff–Bloom–Richardson grade in breast cancer patients; and its high expression correlates with larger tumor size as well as severer tumor invasion in colorectal cancer patients [17, 26, 27]. In brief, these studies imply that CCT6A may be involved in disease initiation and progression in patients with specific cancers. For lung cancer, there is only one investigation shows the correlation of CCT6A with worse survival in NSCLC patients (which is only supported by gene expression analysis in TCGA) [16, 19]. All these data reveal that CCT6A may be dysregulated and be associated with the clinicopathological features of NSCLC patients, while little information was known about CCT6A in cancer progression of NSCLC patients. To solve this problem, we retrospectively screened 381 NSCLC patients and investigated the CCT6A expression in tumor tissue and adjacent tissue. As a result, we found that CCT6A expression was overexpressed in tumor tissue compared with adjacent tissue, and ROC curve showed that CCT6A could distinguish tumor tissue from adjacent tissue (despite some patients presented with CCT6A expression in adjacent tissue, it did not affect the predictive power of CCT6A). The possible reasons for this result might be as follows: as the influence of CCT6A on cellular functions in other cancers, CCT6A might enhance malignant cell proliferation via accelerating the G1-to-S transition, which further facilitated the NSCLC occurrence; thus, increased CCT6A expression was observed in tumor tissue compared with adjacent tissue in NSCLC patients [17, 18]. Furthermore, we also explored the association of tumor CCT6A expression with clinicopathological features and observed that tumor CCT6A high expression was correlated with LYN metastasis, raised TNM stage, and abnormal CEA in NSCLC patients. Compared with the previous study using TCGA, both the previous analysis and our study uncovered the role of CCT6A in NSCLC, while there were remarkable differences between these two studies: the analysis using TCGA dataset mainly focused on the association between exome-wide low-frequency or rare genetic variants and the outcomes of NSCLC patients, while our study disclosed the overexpression of CCT6A in tumor tissues compared with adjacent tissue, the positive correlation of CCT6A with clinicopathological features and the predictive value of CCT6A for worse survival profiles in NSCLC patients.

As to the possible reasons for our findings, these results might be explained as follows: (1) regarding LYN metastasis, CCT6A might enhance the metastasis via promoting TGF-β signaling, thus its high expression correlated with LYN metastasis in NSCLC patients [16]; (2) as for TNM stage, CCT6A might facilitate cell proliferation through promoting G1-to-S phase in NSCLC cells, thereby accelerated tumor growth and led to advanced TNM stage in NSCLC patients [18]; (3) with regard to abnormal CEA, CCT6A might be implicated in the production and release of excess CEA, thus CCT6A high expression correlated with abnormal CEA, while the detailed mechanism remained unclear. Notably, previous studies reveal that the CCT/TCP-1 ring complex (TRiC) chaperonin complex is involved in the protein folding, which may further affect the cellular functions, thereby may fundamentally be related to cancer cell activities [28]. For instance, CCT/TRiC interacts with the tumor suppressor protein von Hippel–Lindau (VHL) in mammalian cells and interferes with VHL function and mutation, which further contributes to the occurrence of various tumors such as sporadic renal clear cell carcinomas [29, 30]; besides, CCT/TRiC mediates the folding and activity of Stat3, which is a crucial regulator preventing apoptosis of parenchymal cells in several acute injuries such as myocardial infarct or traumatic injury [31]; in addition, CCT/TRiC is also well known to mediate the folding of cytoskeletal proteins including tubulins and actins [16, 32]. Considering CCT/TRiC has shown various biological functions, for CCT6A, its exact mechanisms in NSCLC cells remains largely unknown, and continual efforts are needed to explore its detailed molecular mechanisms in NSCLC cells. Furthermore, the study conducted by Ying et al. indicates that CCT6A levels are correlated with a high metastasis potential in patients with high TGF-β levels, whereas in patients with low TGF-β levels, CCT6A expression is not associated with NSCLC metastasis. Indeed, it has been shown that CCT6A promote the survival and metastasis of NSCLC cells in a TGF-β signaling-dependent manner; thus, CCT6A may display a close correlation with metastasis in patients with high TGF-β level, while more explorations are needed to validate this issue [16].

In addition, some studies support the role of CCT6A as a biomarker for survival in prediction in cancer patients [18, 26]. For instance, one study displays that CCT6A high expression is associated with decreased OS in HCC patients [18]. Besides, CCT6A expression is negatively correlated with distant metastasis-free survival, DFS, post-progression survival, and OS in breast cancer patients [26]. However, few studies reveal the predictive value of CCT6A for prognosis in NSCLC patients. Our study found that tumor CCT6A high expression was an independent predictive factor for shorter DFS and OS in NSCLC patients. Noticeably, some other characteristics of NSCLC patients, including poor differentiation, LYN metastasis, higher TNM stage, and abnormal CEA level also had the good ability to distinguish NSCLC tissue from adjacent tissue, and our data indicated that the ability of CCT6A in distinguishing NSCLC tissue from adjacent tissue was better or inferior compared with these biomarkers. The possible reasons were (1) CCT6A promoted cell proliferation and metastasis, which enhanced tumor progression; thus, the CCT6A expression was negatively correlated with DFS and OS in NSCLC patients (as we discussed above) [16, 18]; (2) CCT6A might facilitate drug resistance in NSCLC (as its role in melanoma), which eventually led to unfavorable treatment outcomes; thus, CCT6A high expression predicted worse survival profiles in NSCLC patients [25].

Some limitations existed in our study: (1) this was a single-center study; thus, there might be some selective biases; (2) as a retrospective study, our findings should be validated in a prospective study; (3) as the results show that CCT6A can independently predict LYN metastasis, a metastasis-free survival analysis in further prospective studies would be better to validate our findings; (4) although our study displayed that CCT6A high expression was an independent risk factor for NSCLC patients, it was needed to investigate whether CCT6A was an independent risk factor for all NSCLC clinical case categories of LYN metastasis or only for a specific category; (5) detailed mechanism underlying the role of CCT6A in NSCLC pathology was not investigated in this present study, which was needed to be further explored.

## Conclusion

In summary, CCT6A expression is upregulated in NSCLC tumor tissue compared with adjacent tissue; meanwhile, its high tumor expression correlates with LYN metastasis, increased TNM stage, abnormal CEA, and independently predicts poor DFS as well as OS in NSCLC patients.

## Supplementary information

**Additional file 1: Table S1.** Factors related to LYN metastasis

**Additional file 2: Figure S1.** Study flow

**Additional file 3: Figure S2.** IHC score and ROC curve. Comparison of CCT6A IHC score between tumor tissue and adjacent tissue (**A**). Ability of CCT6A for distinguishing NSCLC tissue from adjacent tissue (**B**). IHC, immunohistochemistry ROC curve, receiver-operating characteristic curve; AUC, area under the curve; CI, confidence interval; CCT6A, chaperonin containing t-complex polypeptide 1 subunit 6A; NSCLC, non-small cell lung carcinoma.

**Additional file 4: Figure S3.** Correlation of several major characteristics of NSCLC patients with survival profiles. Correlation of differentiation (**A**), LYN metastasis (**B**), TNM stage (**C**) and CEA level (**D**) with DFS in NSCLC patients. Correlation of differentiation (**E**), LYN metastasis (**F**), TNM stage (**G**) and CEA level (**H**) with OS in NSCLC patients. NSCLC, non-small cell lung carcinoma; LYN, lymph node; TNM, Tumor Node Metastasis; CEA, carcinoembryonic antigen; DFS, disease-free survival; OS, overall survival.

## Data Availability

All data generated or analyzed during this study are included in this published article.
