# Supplementary material for: Chaperonin containing t-complex polypeptide 1 subunit 6A correlates with lymph node metastasis, abnormal carcinoembryonic antigen and poor survival profiles in non-small cell lung carcinoma
Source: World J Surg Oncol. 2020 Jul 6;18:156. doi: 10.1186/s12957-020-01911-x (PMC7339415; doi:10.1186/s12957-020-01911-x)
Supplement: Supplementary file 1 — Additional file 1: Table S1. Factors related to LYN metastasis [file 12957_2020_1911_MOESM1_ESM.docx]

| Items | Multivariate logistic regression model | | | |
| --- | --- | --- | --- | --- |
|  | *P* value | OR | 95%CI | |
|  |  |  | Lower | Higher |
| CCT6A high | 0.012 | 1.826 | 1.140 | 2.925 |
| Age (>60.0 years) | 0.027 | 1.713 | 1.063 | 2.761 |
| Male | 0.330 | 0.761 | 0.438 | 1.320 |
| History of smoke | 0.721 | 0.917 | 0.569 | 1.477 |
| History of drink | 0.487 | 1.187 | 0.732 | 1.925 |
| Hypertension | 0.593 | 1.168 | 0.661 | 2.064 |
| Hyperlipidemia | 0.651 | 0.871 | 0.478 | 1.586 |
| Diabetes | 0.744 | 0.894 | 0.455 | 1.755 |
| Poor differentiation | 0.346 | 1.201 | 0.820 | 1.759 |
| Tumor size (>5.0 cm) | <0.001 | 4.678 | 2.876 | 7.612 |
| Abnormal CEA (>5.0 ng/mL) | 0.026 | 1.743 | 1.067 | 2.846 |
