# Supplementary figures and images for: Chaperonin containing t-complex polypeptide 1 subunit 6A correlates with lymph node metastasis, abnormal carcinoembryonic antigen and poor survival profiles in non-small cell lung carcinoma
Source: World J Surg Oncol. 2020 Jul 6;18:156. doi: 10.1186/s12957-020-01911-x (PMC7339415; doi:10.1186/s12957-020-01911-x)

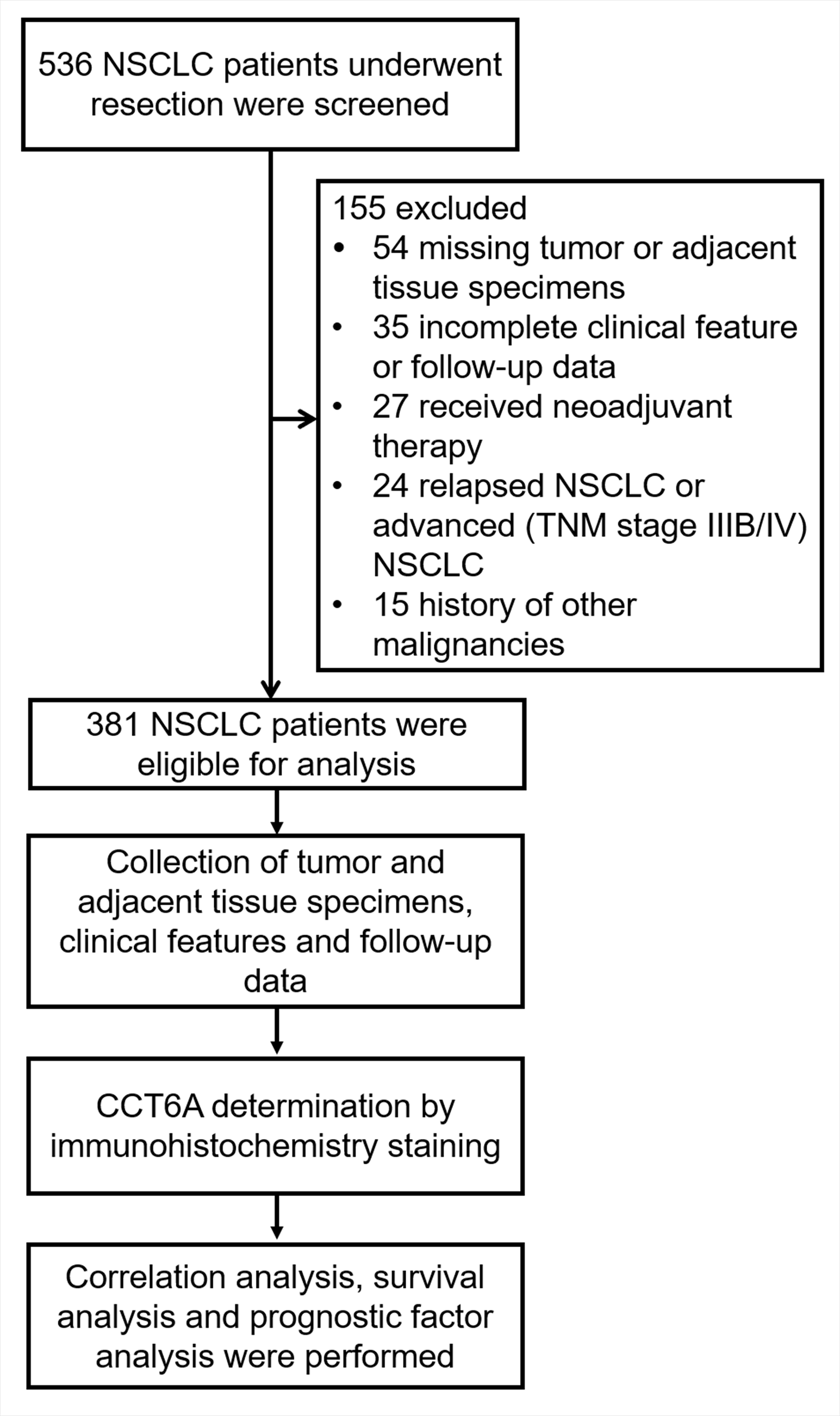

Supplement: Supplementary file 2 — Additional file 2: Figure S1. Study flow [file 12957_2020_1911_MOESM2_ESM.tif]

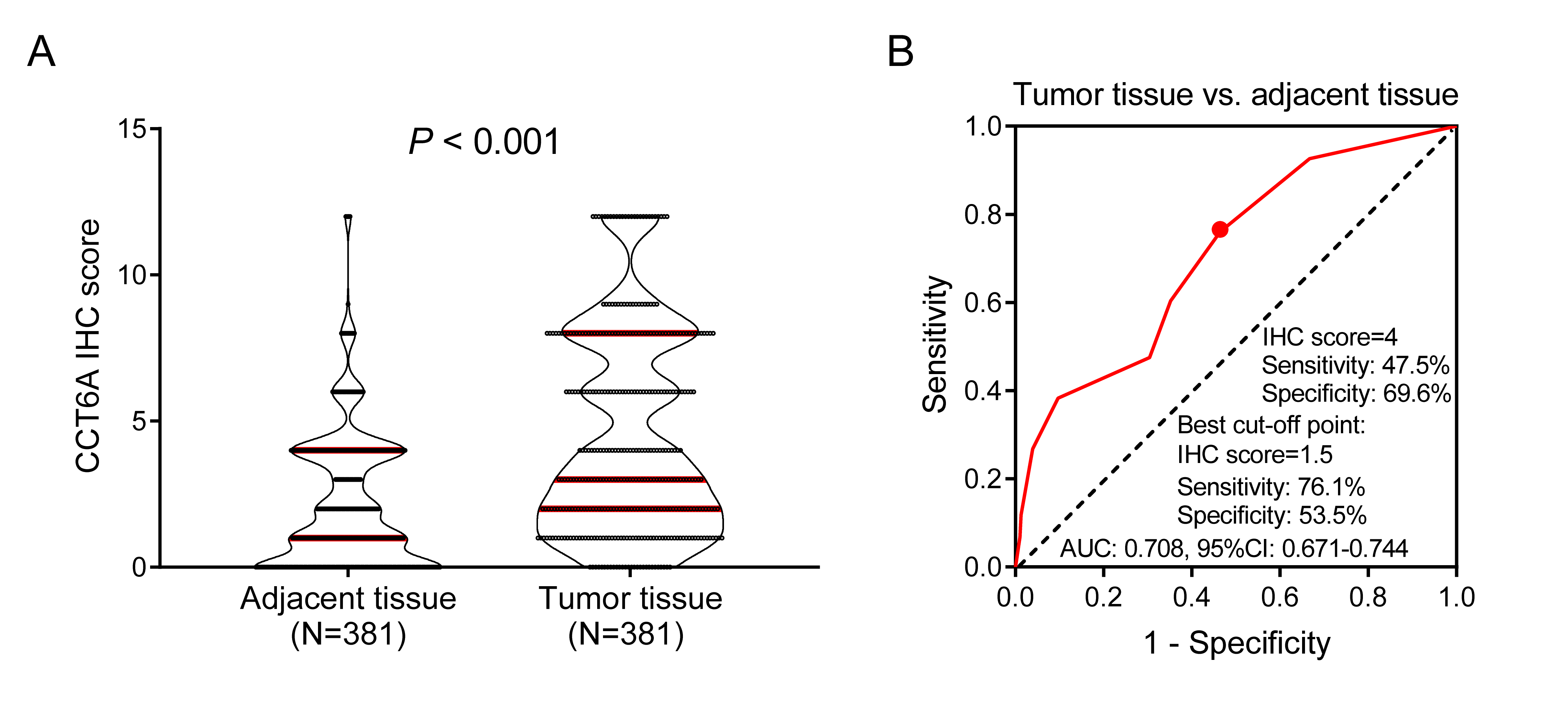

Supplement: Supplementary file 3 — Additional file 3: Figure S2. IHC score and ROC curve. Comparison of CCT6A IHC score between tumor tissue and adjacent tissue (A). Ability of CCT6A for distinguishing NSCLC tissue from adjacent tissue (B). IHC, immunohistochemistry ROC curve, receiver-operating characteristic curve; AUC, area under the curve; CI, confidence interval; CCT6A, chaperonin containing t-complex polypeptide 1 subunit 6A; NSCLC, non-small cell lung carcinoma. [file 12957_2020_1911_MOESM3_ESM.tif]

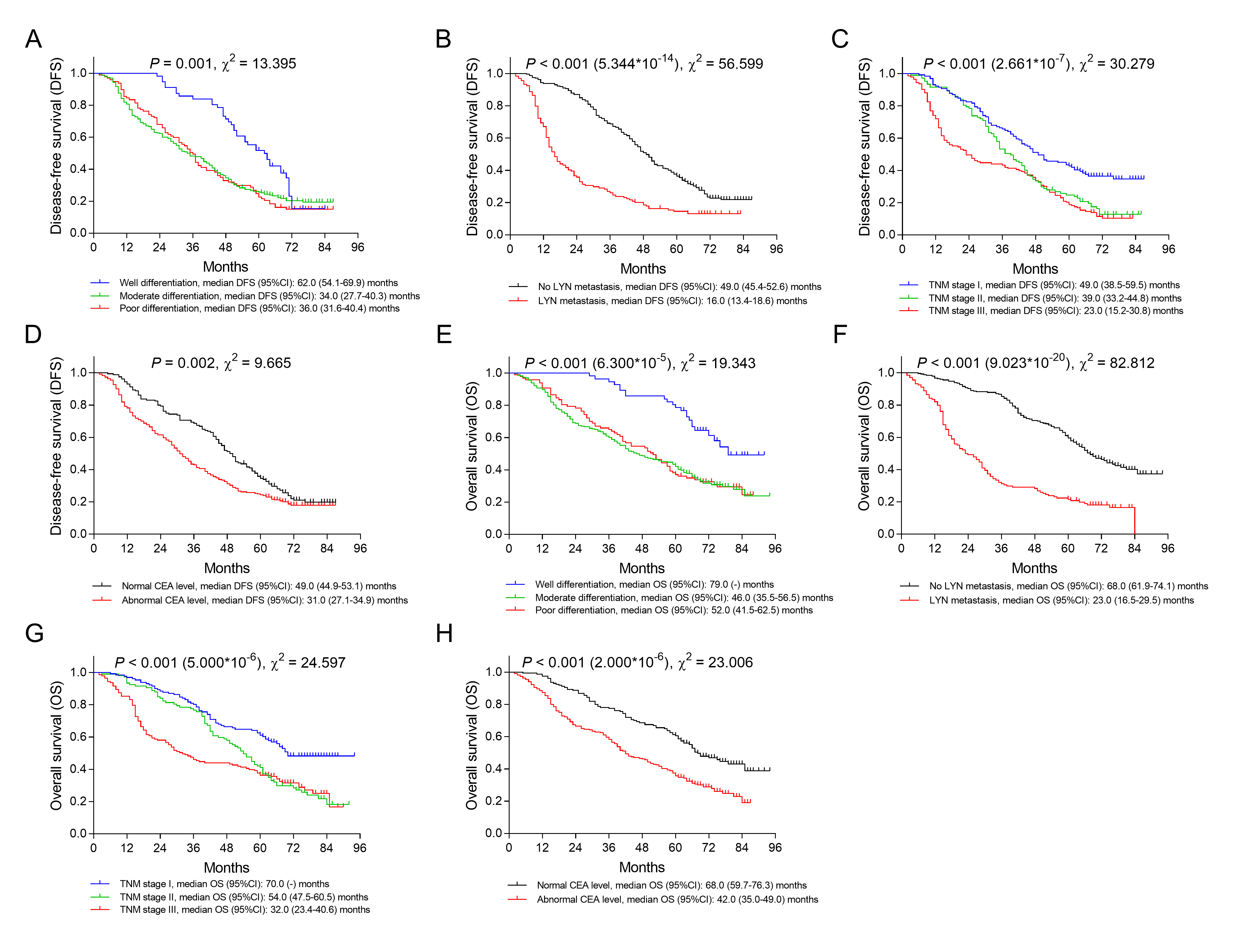

Supplement: Supplementary file 4 — Additional file 4: Figure S3. Correlation of several major characteristics of NSCLC patients with survival profiles. Correlation of differentiation (A), LYN metastasis (B), TNM stage (C) and CEA level (D) with DFS in NSCLC patients. Correlation of differentiation (E), LYN metastasis (F), TNM stage (G) and CEA level (H) with OS in NSCLC patients. NSCLC, non-small cell lung carcinoma; LYN, lymph node; TNM, Tumor Node Metastasis; CEA, carcinoembryonic antigen; DFS, disease-free survival; OS, overall survival. [file 12957_2020_1911_MOESM4_ESM.tif]
